# Ultrasound analysis of the masseter and anterior temporalis muscles in edentulous patients rehabilitated with full-arch fixed implant-supported prostheses

**DOI:** 10.1007/s00784-024-05676-5

**Published:** 2024-05-09

**Authors:** Bahar Alkaya, Hazal Duyan Yüksel, Burcu Evlice, Mustafa Özcan, Onur Uçak Türer, Sıla Çağrı İşler, Mehmet Cenk Haytaç

**Affiliations:** 1 Department of Periodontology Faculty of Dentistry, Cukurova University, Adana, Turkey; 2 Department of Oral Diagnosis and Maxillofacial Radiology, Faculty of Dentistry, Cukurova University, Adana, Turkey; 3 Department of Periodontology, School of Dental Medicine, Bern University, Bern, Switzerland; 4 Department of Periodontology, Faculty of Dentistry, Gazi University, Ankara, Turkey

**Keywords:** Masseter muscle thickness, Implant-supported fixed prostheses, Ultrasonography

## Abstract

**Objectives:**

Total tooth loss is common in the aging population resulting in insufficient chewing function with subsequent weakening of the masticatory muscles. The study aims to evaluate the changes in thicknesses of the masseter and anterior temporal muscle in edentulous patients following the reconstruction of implant-supported fixed prostheses and compare them with the dentate individuals.

**Materials and methods:**

The study was designed as a prospective, single-center, controlled clinical trial. A total of 60 participants were included in the present study. The patients were divided into two groups; Group I (Test Group): 30 edentulous patients who received implant-supported fixed prostheses, Group II (Control Group): 30 dentate individuals of an age and sex-matched group. Ultrasonography was used to measure the cross-sectional thickness of the left and right musculus masseter and anterior temporalis immediately after the cementation of the prosthetic rehabilitation (T1), on the 1st (T2) and 6th (T3) months after rehabilitation and at a single time point in the control group.

**Results:**

The results showed that there were significant comparison differences in muscle thickness at the baseline measurements between groups while at the end of the 6th month, these differences were not significant. The muscle thicknesses of both the masseter and anterior temporalis muscles increased significantly at T2 and T3 compared to T1 in the test group. The asymmetry index between the left and right muscles in the test group and the asymmetry differences between groups also decreased significantly at the end of the 6th month.

**Conclusion:**

The implant-supported fixed prostheses significantly increase the thicknesses of the masseter and anterior temporal muscle together with a decrease in the asymmetry between the left and right muscles. At six months, implant-treated patients showed similar muscle thicknesses compared to dentate individuals.

**Clinical relevance:**

The findings suggest that implant-supported fixed prostheses can improve the masticatory function and facial symmetry of edentulous patients.

## Introduction

According to the World Health Organization, total edentulism (complete loss of all permanent teeth) is an important disability affecting a significant population [1]. Tooth loss influences the connection between oral and facial tissues, the jaw joint, muscle structure, and the nervous system [2–4]. Occlusal strength and masticatory performance can be decreased because of tooth loss [3, 5]. This condition has negative effects on the dietary regimens of patients [6, 7]. Numerous studies have shown the correlation between edentulism and malnutrition [8–10]. Reduced masticatory function results in a decrease in muscle fiber size, causing muscle atrophy and morphological changes [11]. The masseter muscle thickness (MMT) decreases as a result of tooth loss [12, 13].

Complete edentulous arches can be treated with implant-supported fixed bridges, implant-supported overdentures, or conventional complete prosthetic rehabilitation [3, 14]. Conventional complete dentures have been the primary treatment option for edentulous patients for many years [15]. However, the developments in dental materials and dental technology sciences have revolutionized treatment options for edentulous individuals with implant-supported fixed and/or retained prostheses [15]. Implant treatments are preferred more often among edentulous patients compared to conventional complete dentures due to their ability to provide better stability, increased chewing performance, and a more permanent solution [16, 17]. As the number of the placed implants increases, the stability of the prosthesis will also increase. Therefore, the ideal contemporary treatment protocol for all edentulous patients is implant-supported fixed reconstruction [18, 19]. The most important aim of edentulous patient treatment is the restoration of their chewing and muscle function to be able to make it closer or the same as dentulous ones [20, 21].

The masticatory muscles’ thickness has been widely regarded as the primary determinant contributing to bite force in the adult population [22]. The four main muscles used for chewing are the masseter, medial–lateral pterygoid, and temporalis muscles. The masseter is the mandible’s primary elevator muscle. In the closing action of the lower jaw, three muscles work together: the pterygoid muscle contributes about 21%, the temporalis muscle contributes about 36%, and the masseter muscle contributes about 43% to the total jaw-closing muscle strength [23]. Previous studies have shown that masseter muscle thickness has a strong relationship with the size of the bite force, and measuring muscle thickness is considered a reliable method to assess muscle function [21, 22, 24].

Various non-invasive imaging methods, including electromyography, computed tomography, magnetic resonance imaging (MRI), and ultrasound scanning (US), can be employed to quantify the cross-section and human masticatory muscles’ thickness in vivo [24–26]. Ultrasonography is a practical follow-up method that is reproducible, inexpensive, and easier to use without ionizing radiation [25]. At the same time, it does not have any cumulative biological effects [26]. The most evident disadvantage of the technique is that it allows only superficial muscles to be examined. The masseter muscle’s superficial location provides easy access for ultrasound applications; therefore, it is the most suitable muscle for scanning [27]. Due to these advantages, masseter muscle thickness can be accurately measured by the ultrasound technique [2].

Masseter muscle thickness plays an important role in mastication and facial aesthetics [28]. Asymmetries are common and have important consequences in the human body. Evaluating the craniofacial complex’s functional symmetry of the craniofacial complex (the skull and face) typically involves modeling masticatory movements (chewing) and analyzing muscle activity [29]. Up to date, indexes such as Naeije et al.’s asymmetry index are used to evaluate the symmetry of muscle thickness of muscle in homologous muscles from each side of the body [30].

It is known that the muscle thickness of masticatory muscles changes due to complete edentulism. The current study aims to evaluate the changes in thicknesses of the masseter and anterior temporal muscle in edentulous patients following the reconstruction of implant-supported fixed prostheses and compare these changes with dentated individuals. The null hypothesis of this study is that there will be no changes in muscle thicknesses in edentulous patients after using implant-supported fixed prostheses.

## Materials and methods

The study received approval by the Çukurova University Faculty of Medicine Clinical Research Ethics Committee (Meeting No: 136, Decision No:63). The study was performed in the Periodontology Department at Cukurova University (Adana, Turkey) between March 2021-June 2023 following the guidelines of the 2008 Helsinki Declaration. The participants were provided with a detailed explanation of the study approach, and their agreement was obtained through both verbal and written means.

## Sample size calculation

The determination of the sample size was conducted using the G*Power 3.1 program. As there were no previous studies with a similar design in the literature to our knowledge, the medium effect size suggested by Cohen (Cohen’s d = 0.25) was used, and the sample size for the study and control groups was calculated as approximate sample size of n:28 (α: 0.05, β: 0.80). Since it was a follow-up study, the final sample size for the groups was determined as 30 and the total sample size as 60 in order to compensate possible drop-outs.

### The study population

Based on dental status, the sample selected was divided into two main groups. Test group consisted of completely edentulous patients following the reconstruction of implant-supported fixed prostheses. Age and gender-matched control group (CG) was recruited from persons who were referred to the clinic for general periodontal prophylaxis. This group consisted of persons who had only a maximum of one missing posterior tooth with no previous history of periodontal bone loss or bruxism.

The patients with complete edentulism were selected to compare the masseter and anterior temporal muscle cross-sectional thickness changes during static and dynamic activities immediately after the placement and at the 1st and 6th months of implant-supported fixed prostheses use. A comparison was also made between the test and control groups to evaluate the effect of loss of dentition on the masseter and anterior temporalis muscle thicknesses.

#### Inclusion criteria

- Systemically healthy.
- Non-smokers.
- Dentate individuals were selected with a maximum one missing posterior tooth.
- Edentulous individuals who were at least 3 months and a maximum 6 months of total edentulism.
- Edentulous individuals who have never used prosthesis before.
- No contraindications to implant placement.

#### Exclusion criteria

- The presence of muscle disorders.
- Myopathy in the head, neck, or shoulders.
- Individuals who have parafunctional habits including bruxism and clenching.
- Individuals having any kind of temporomandibular disorders.

At the end, 60 patients with similar age (test group mean 51.8 ± 9 (36–67) vs. control group mean 49.2 ± 9.1(32–63)) and gender distribution (study group 15 female,15 male; control group 15 female,15 male) were included in the study.

### Clinical procedures

The implant planning was done by baseline cone beam computed tomography to evaluate the bone level, quality, and location of anatomical landmarks for edentulous patients (test group). All tomographs were obtained with calibrated and standardized main radiological characteristics (exposure parameters: 90 kV, 10 mA, 27 s scan time; voxel size: 0.4 mm).

Six or seven dental implants were surgically inserted into the edentulous maxilla and mandible (total 12–14 implants) of each patient. All surgical procedure was performed according to standard procedures. There were no surgical models used. All surgeries were performed under local anesthesia (Ultracine D-S Fort Adrenaline®, İstanbul, Türkiye). The residual alveolar bone’s shape and anatomical markers determined the number and location of the implants. The angulations of the implants were checked with parallel pins during the surgery. The implants were inserted with 1–2 mm below the level of the alveolar crest to improve primary implant stability. Implants were closed with a surgical closure screw. The sutures were removed after 10–12 days. After the operation, patients received 2 g of penicillin twice a day for 10 days or in case of penicillin allergy, clindamycin 300 mg every 6 h for 7 days. All patients rinsed their mouths twice a day for 10 days with an antimicrobial chlorhexidine solution. They were advised to eat a soft, nutritious diet. After 3 months of healing and the osseointegration phase, healing abutments were placed and then the fixed implant-supported prosthesis stage started. Intercuspal-centered contacts were ensured to be evenly distributed among all occlusal systems. The prostheses were checked in terms of aesthetics, centric relationships, and occlusion. The prosthetic rehabilitation was cemented with polycarboxylate cements on the abutments.

Muscle thickness measurements for the test group were performed on three different periods and at a single time point for the control group. Ultrasonography was conducted to assess their masticatory muscles (masseter and anterior temporalis) immediately after the cementation of the prosthetic rehabilitation (T1), on the 1st (T2) and 6th (T3) months after rehabilitation. The cross-sectional thickness of the left and right musculus masseter and anterior temporalis was recorded for all individuals. The primary outcome of this study was the dimensional changes in muscle thickness measured over three different periods in the same patient and a comparison of the results with dentate individuals.

### Ultrasonographic evaluation

The assessment thickness of the masseter and anterior temporalis muscles was bilaterally measured using a USG device (B-mode, 7 MHz ultrasound scanner and probe (linear), Clarius Mobile Health, Vancouver, Canada). The same radiologist performed all scanning and measurement procedures (HDY). The calibration of the researcher was assessed by a baseline test of 10 volunteers who were not enrolled in this study. Ultrasonographic measurements of these volunteers were recorded at 1-week intervals. The intra-class correlation of coefficient (ICC) was calculated for intra-observer reproducibility for muscle thickness measurement to verify calibration and consistency. The ICC value was equal to 0,93 (0.73–0.98 = confidence interval of 95% ) and excellent agreement was observed (*p* < 0.001).

**Fig. 1:**
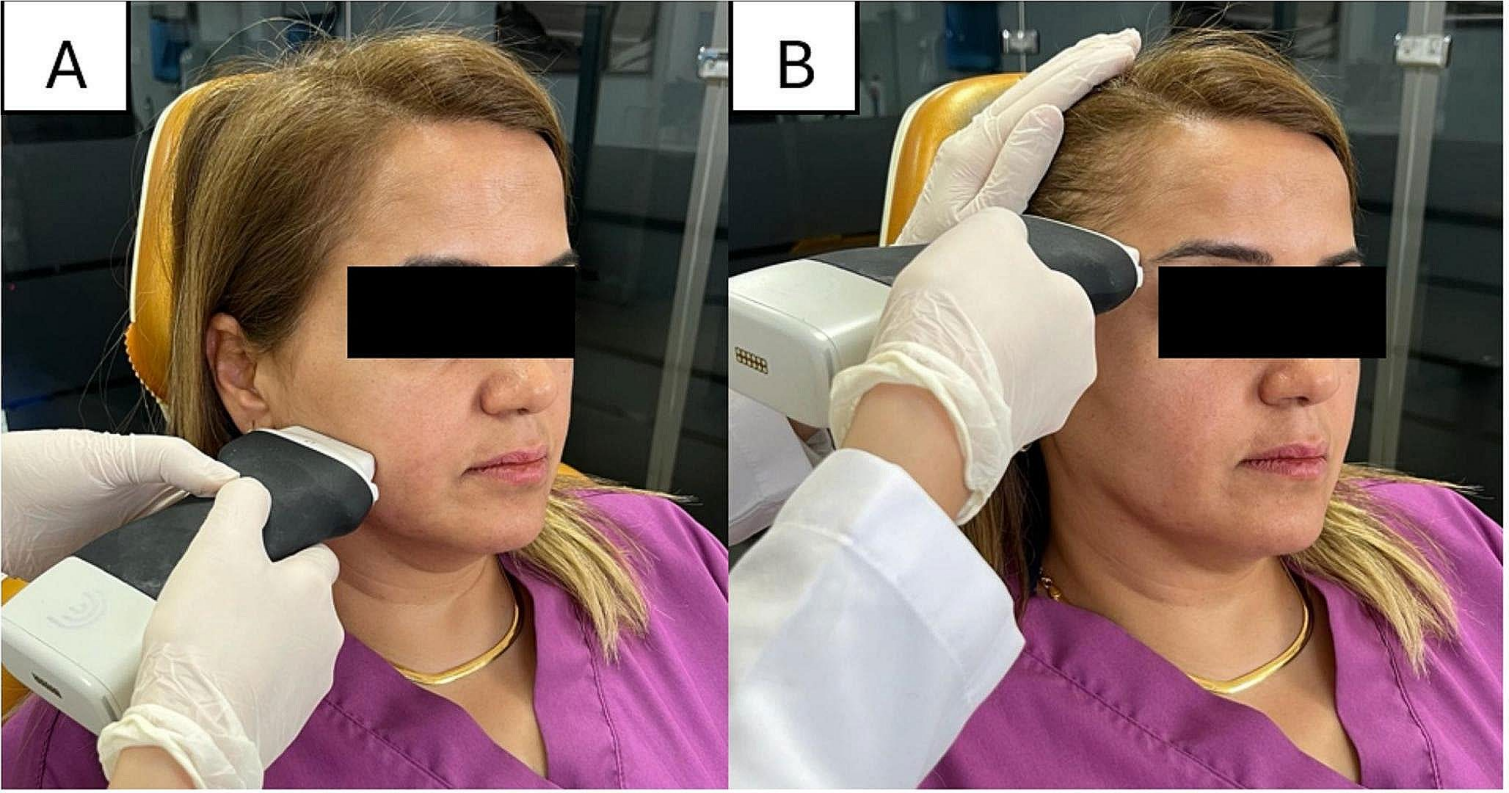
Positioning of the USG probe for the masseter (**A**) and anterior temporalis (**B**) muscle measurements

**Fig. 2:**
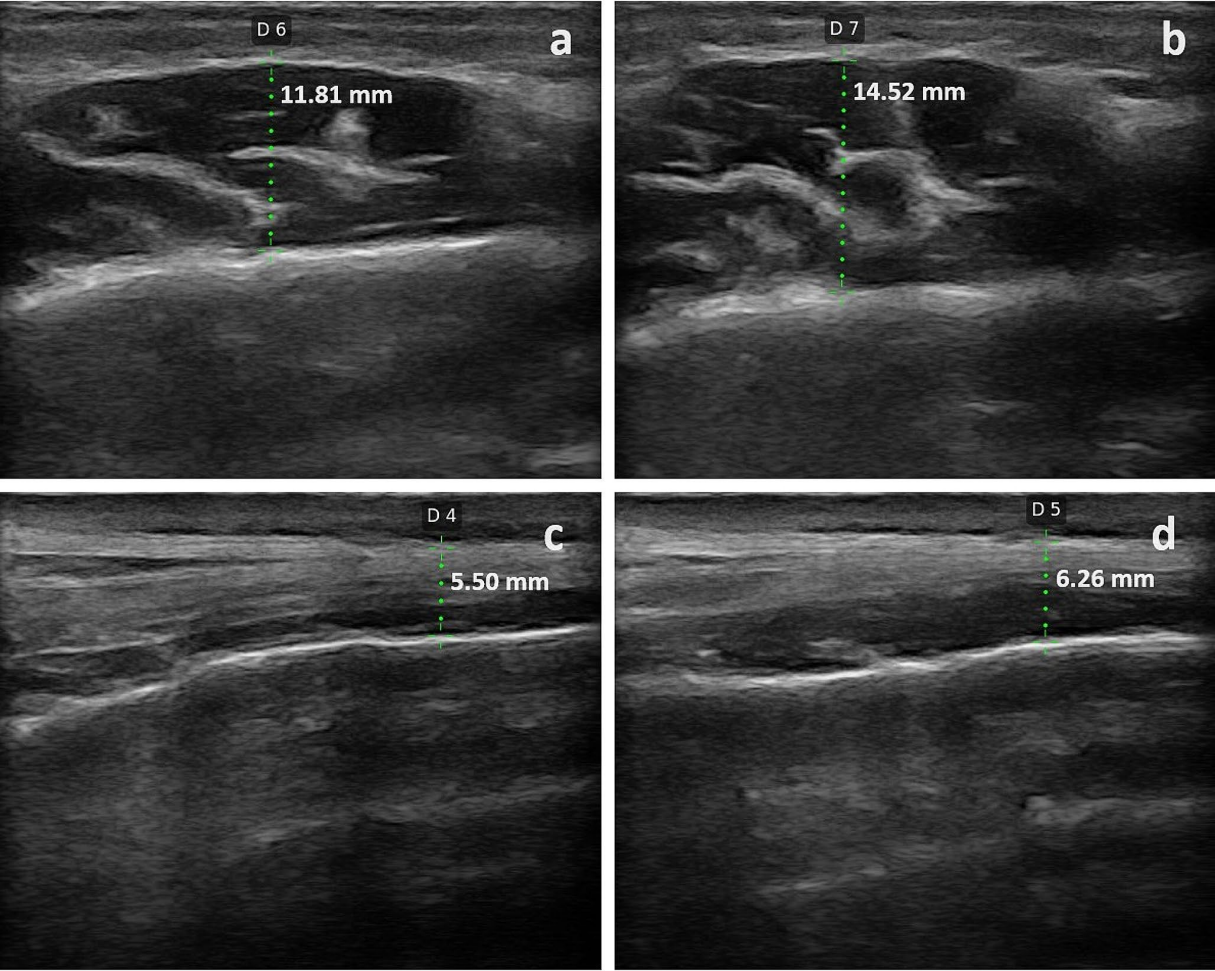
Cross-sectional thicknesses of the masseter and anterior of temporalis muscles. (**a**) Relaxed state masseter (**b**) Contracted state masseter (**c**) Relaxed state anterior of temporalis (**d**) Contracted state anterior of temporalis

The patients were positioned in such a way that the Frankfurt horizontal plane was maintained parallel to the floor during the imaging process. (Fig. 1) A water-soluble transmission gel was used between the probe and the skin to effectively transmit sound waves. USG measurements were analyzed for right-left m. masseter and right-left m. anterior temporal muscles in both the contracted (maximum bite) and the relaxed positions in each patient. (Fig. 2)

The resting state of the masseter muscle is characterized by the mouth being slightly open, approximately 8–9 mm, while exhibiting minimal electromyographic activity. Contraction of the muscle occurs when the jaws close and the teeth come into contact. The assessment of the thickness of the masseter muscle was conducted at the maximum width between the lateral side of the mandibular ramus and the muscle’s external fascia, specifically in the middle third of the mandible’s ramus. One centimeter above the zygomatic arch, parallel to the temporal bone, and perpendicular to it, the temporal muscle’s anterior part was examined. This particular site is situated directly anterior to the hairline’s anterior boundary.

Symmetrical changes in muscle thickness were measured on both the left and right sides. Up to our knowledge, there is no existing formula to detect the asymmetry of muscle thicknesses in the literature however, the following formula was developed by Naeije et al. to calculate the variation in muscle activity between right and left sides.

$$\begin{array}{l}{\rm{Asymmetry}}\,{\rm{index}}\,{\rm{ = }}\\\,\,\,\,\,\,\,\frac{{{\rm{Right}}\,{\rm{masseter}}\,{\rm{activit}}{{\rm{y}}^{\rm{ - }}}\, - \,{\rm{Left}}\,{\rm{masseter}}\,{\rm{activity}}}}{{{\rm{Right}}\,{\rm{masseter}}\,{\rm{activity}}\,{\rm{ + }}\,{\rm{Left}}\,{\rm{masseter}}\,{\rm{activity}}}} \times 100\end{array}$$

In the current study, this original formula was modified to use the muscle thickness measurements instead of the muscle activity to evaluate the asymmetry of the muscles.

Each measurement was carried out three times at five-minute intervals to minimize measurement errors. The thickness used for statistical data analysis was determined by calculating the average of the three measurements on each side. Patients were given rest between data collection periods to prevent muscle fatigue. The same measurement protocol was repeated at each evaluation period for the test group.

### Statistical analysis

The IBM SPSS Statistics 20 (Armonk, NY: IBM Corp.) program was used for statistical analysis. A level of significance of 0.05 was used for all tests. A summary of quantitative variables was provided using mean ± standard deviation, frequency (%), or median (maximum- minimum). Shapiro-Wilk tests were utilized to confirm the distribution’s normality. Time-dependent changes in muscle thickness measurements and asymmetry index were examined by the Friedman test. For pair-wise comparisons of time-dependent changes, the Wilcoxon signed-rank test with *p*-value correction was used.

Mann-Whitney U test was used for comparisons between the muscle thicknesses of the control group and the study group at each time point.

## Results

A total of 60 patients were enrolled in this study, and all patients completed the study. There were no statistical differences in terms of age (mean test group 51.8 ± 9 (36–67); mean control group 49.2 ± 9.1(32–63) *p* = 0.278)) and gender distribution (study group 15 female,15 male; control group 15 female,15 male) between groups.

Table 1 presents a summary of the average cross-sectional thickness of the masseter and anterior temporalis muscles in both the relaxed and contracted positions, for both the left and right sides for the control and test group (prosthesis insertion, 1st and 6th month post-insertion). There was a statistically significant difference in anterior temporal muscle thickness at both T1 and T2 time points (*p* < 0.05) between the test and controls. MMT in the test group was statistically significantly lower than in the control group at baseline (*p* < 0.001), but this difference was not present at T2 and T3 time points. MMT was measured even slightly higher in the test group (although not statistically significant) than the controls at the end of the 6th month. The average of all muscle thickness measurements showed statistically significant increases in both the contracted and the relaxed positions from baseline to the 1st and to 6th months in test group. This increasing trend was significant for all muscle thickness measurements in both resting and contracted states from baseline to the 1st month (*p* < 0.001), baseline to the 6th month (*p* < 0.001), and from the 1st month to the 6th month (*p* < 0.001).

**Table 1:** Masseter and anterior temporalis muscle cross-sectional thickness in relaxed and contracted states at left and right sides for control and test group (prothesis insertion, 1st and 6th month post-insertion)

Side	Position	Control Group	Test Group baseline (T1)	Test Group 1st month (T2)	Test Group 6th month (T3)	*p*^1^ value within Test Group^a^	*p*^2^ value Control vs. Test baseline^b^	*p*^3^ value Control vs. Test 1st month^b^	*p*^4^ value Control vs. Test 6th month^b^
Right Masseter	RP	11.3 ± 1.04 11.3 (9.31–13.61)	8.2 ± 2.02 ^φ,Ψ^ 7.98 (5.15–12.46)	10.91 ± 1.8^,Ψ^ 10.58 (7.3-14.18)	12.17 ± 1.76 11.7 (10.32–15.5)	< 0,001*	< 0.001*	0.193	0.124
	CP	13.71 ± 1.39 13.44 (11.03–16.57)	9.69 ± 2.26 ^φ,Ψ^ 9.9 (5.56–13.75)	13.13 ± 1.49^,Ψ^ 12.98 (10.17–15.93)	14.46 ± 1.48 14.76 (12.41–16.6)	< 0,001*	< 0.001*	0.167	0.101
Left Masseter	RP	11.63 ± 1 11.53 (10.01–13.46)	9.37 ± 2.14 ^φ,Ψ^ 8.78 (5.3–13.8)	11.03 ± 2.3^,Ψ^ 11.29 (4.25-15)	12.07 ± 1.85 11.97 (8–15)	< 0,001*	< 0.001*	0.209	0.105
	CP	13.8 ± 1.4 13.39 (10.69–16.09)	10.74 ± 1.93 ^φ,Ψ^ 10.66 (6.87–14.23)	13.11 ± 1.93^,Ψ^ 12.96 (7.11–16.33)	14.42 ± 1.51 15 (12-16.6)	< 0,001*	< 0.001*	0.117	0.147
Right Anterior Temporalis	RP	7.53 ± 0.87 7.62 (6.21-9)	5,11 ± 1,32 ^φ,Ψ^ 4,92 (3,13 − 7,59)	6,42 ± 1,07^,Ψ^ 6,4 (4,99 − 8,2)	7,21 ± 1,04 7,35 (5,91 − 9,55)	< 0,001*	< 0.001*	< 0.001*	0.121
	CP	8.66 ± 1.2 8.44 (7.02–11.03)	5,94 ± 1,34 ^φ,Ψ^ 5,91 (3,84 − 8,26)	7,37 ± 1,1^,Ψ^ 7,64 (5,1–8,89)	8,17 ± 1,05 8,17 (5,98 − 9,69)	< 0,001*	< 0.001*	< 0.001*	0.271
Left Anterior Temporalis	RP	7.99 ± 0.9 8.2(6.51–9.69)	5,9 ± 1,3 ^φ,Ψ^ 5,85 (4,06–8,4)	7,19 ± 1,02^,Ψ^ 7,43 (5,39 − 8,78)	7,97 ± 1,16 8,17 (5,41 − 9,84)	< 0,001*	< 0.001*	< 0.007*	0.988
	CP	8.97 ± 1.23 9.01 (7.02–11.03)	6,78 ± 1,22 ^φ,Ψ^ 6,77 (4,68 − 8,74)	8,04 ± 0,97^,Ψ^ 7,66 (6,49 − 9,97)	8,87 ± 1,09 9 (7–11,03)	< 0,001*	< 0.001*	< 0.002*	0.574

RP: Rest position CP: Contracted position

^a^Friedman test (p^1^) ^φ,Ψ^ Significant difference for pairwise comparisons - Wilcoxon signed-rank test, ^φ^ Compared to 1st month. ^Ψ^ Compared to 6th month

^b^Mann Whitney U testi. (p^2^ compared to CG with SG baseline, p^3^ compared to CG with SG 1st month, p^4^ compared to CG with SG 6th month)

*p < 0.05

Left masseter muscle thicknesses were generally greater than right masseter muscle thicknesses in both contraction and resting positions at initial T1 measurements in test group. Asymmetry with a left-sided predominance was observed. The asymmetry ratios of the masseter muscle thickness decreased statistically from baseline to the 1st month (*p* < 0.05) and from baseline to the 6th month (*p* < 0.05), while there were no statistically significant differences in asymmetry change between the 1st and 6th months (*p* > 0.05). Intergroup comparisons of the asymmetry index for masseter muscles yielded similar results, such as muscle thickness changes. There was a statistical difference between the groups at T1(*p* < 0.05), no difference was observed at T2 and T3. (*p* > 0.05) (Table 2).

**Table 2:** Asymmetry index changes

Side	Position	Control Group	Test Group baseline (T1)	Test Group 1st month (T2)	Test Group 6t^h^ month (T3)	*p*^1^ value within Test Group	*p*^2^ value Control vs. Test baseline	*p*^3^ value Control vs. Test 1st month	*p*^4^ value Control vs. Test 6th month
Masseter	RP	-1.44 ± 4.02 -0.79 (-17.65-2.29)	-6.77 ± 5.98 ^φ,Ψ^ -6.83 (-19.68-7.07)	0.02 ± 9.83 -0.59 (-22.93-36.92)	0.53 ± 6.58 -0.02 (-9.8-12.85)	0.001*	< 0.001*	0.756	0.906
	CP	-0.32 ± 1.23 -0.34 (-2.76-1.62)	-5.67 ± 7.78 ^φ,Ψ^ -4.94 (-25.26-6.14)	0.36 ± 7.06 -0.92 (-10.83-17.69)	0.16 ± 1.8 0.09 (-3.29-3.78)	0.007*	< 0.001*	0.469	0.220
Anterior Temporalis	RP	-3.01 ± 5.72 -0.57 (-21.39-0.83)	-7,58 ± 8,21 -5,43 (-18,72 − 6,40)	-5,81 ± 5,71 -5,04 (-14,64 − 2,88)	-4,96 ± 5,45 -4,33 (-15,36 − 4,59)	0.356	< 0.001*	0.030*	0.124
	CP	-1.78 ± 3.6 -0.05 (-12.74-3.64)	-6,98 ± 5,35 -6,5 (-17,98 − 0)	-4,55 ± 6,06 -4,95 (-18,94 − 6,36)	-4,16 ± 6,37 -5,06 (-15,92 − 9,53)	0.531	< 0.001*	0.047*	0.086

RP: Rest position CP: Contracted position

^a^Friedman test ^φ,Ψ^ Significant difference for pairwise comparisons (Wilcoxon signed rank test). ^φ^ Compared to 1st month. ^Ψ^ Compared to 6rd month *p < 0.05

Mann Whitney U testi. *p < 0.001. (p^2^ compared to CG with SG baseline, p^3^ compared to CG with SG 1st month, p^4^ compared to CG with SG 6th month)

RP: Rest position CP: Contracted position

**Fig. 3:**
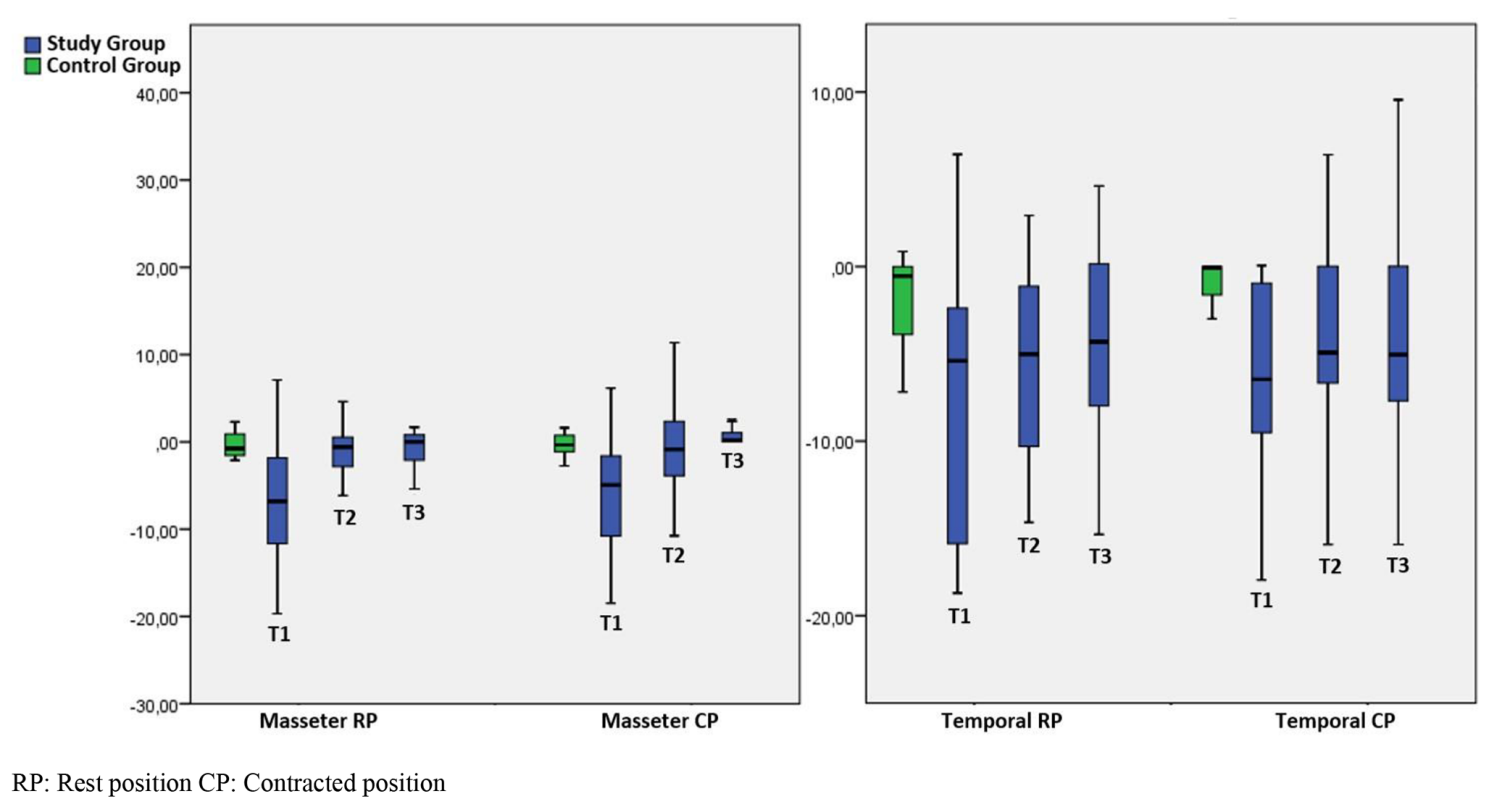
Boxplot graph for the asymmetry index changes in time

The asymmetry of the temporal muscle thicknesses between the right and left sides decreased over time in both contraction and resting positions, although this decrease was not statistically significant. However, statistically significant differences were observed at T1 and T2 in the comparison between groups. (*p* < 0.05) (Table 2) (Fig. 3).

## Discussion

The current study showed significant increases in masseter and temporal muscle thicknesses, both in contraction and at rest, following the placement of fixed implant-supported prostheses in edentulous patients reaching similar muscle thicknesses of dentate individuals at six months. Thus, the null hypothesis was rejected. In addition, the asymmetry difference between the left and right masseter muscle thicknesses decreased significantly over time in the test group.

Implant-supported prostheses offer several advantages compared to conventional removable prostheses, such as increased longevity, improved function, better stabilization and retention, psychologically easier acceptance by patients, and more aesthetic results. In addition, it has been reported that the decrease in masticatory efficiency [31] and biting force due to tooth loss is corrected over time with implant-supported prostheses [32]. It has been shown that biting force is associated with muscle activity [16]. The activity level of the masseter muscle can be directly inferred from its thickness: the more active the muscle, the greater the muscle thickness [33]. In the present study, the increase in muscle thicknesses from baseline to the 6th month in the test group may be attributed to improved masticatory function after rehabilitation. This is consistent with the aim of fixed treatment, which is to provide rehabilitation as similar as possible to dentulous individuals. In inter-group comparisons, there was a statistical difference for both masseter and temporal muscle thicknesses at T1, and a difference only in temporal muscle thickness at T2. This can be explained by the different speeds of neuromuscular adaptation of the muscles.

Prolonged periods of low muscle activity or underutilization can lead to atrophy of the muscles, both at the macroscopic and microscopic levels. The cross-sectional area of the masticatory muscles in edentulous patients is notably reduced in comparison to dentulous patients [34]. In this study, higher thicknesses were measured in the control group than in the test group at the beginning, which supports the literature. Bhoyar et al. observed that the thickness of the masseter muscle was substantially thinner in a population of edentulous participants aged 44–55 years compared to dentulous ones [22]. The same study also found that while complete dentures can help to increase the masseter muscle’s thickness, they do not fully restore it to the same thickness as in dentulous patients. In a study by Müller et al., implant-supported prostheses were found to be related to higher masseter muscle thickness and chewing efficiency than conventional total prostheses in edentulous patients. In the same study, although implant-supported fixed prostheses were associated with higher chewing efficiency than overdenture prostheses, the masseter muscle thickness was the same in both groups and lower than fully dentate participants [34]. In contrast to this finding, masseter muscle thickness was even thicker in the test group at the end of six months compared to dentate individuals in the present study.

Goncalves et al. used three types of prostheses (conventional removable dental prostheses, implant-supported removable dental prostheses, or partial implant-fixed dental prostheses) for 2 months each in 12 partially edentulous patients, in their study [35]. Masseter and temporal muscle thicknesses during rest and maximal clenching were evaluated by ultrasonography. In the study, the chewing efficiency was statistically higher in the group using fixed-supported implants. Although the masseter muscle was thickest in the fixed-supported implant group, no statistically significant difference existed between the groups.

There are only a few studies available so far in the literature examining possible correlation between masseter muscle thickness and its EMG activity [36–38]. Georgiakaki et al. have found that muscle thickness was strongly correlated to electromyographic maximum activity in the masseter muscles while Oliveria et al. did not find significant correlations between thickness and activity of masseter muscles [36, 37]. Comparing edentulous patients treated with immediate loading fixed implant-supported prostheses with dentate patients, similar EMG activity in the masseter muscle was discovered by Mostoyei et al. to that of dentate patients immediately after loading [20]. After 6 months of follow-up, no change in results was observed. The researchers interpreted this finding as evidence that the restoration of masseter muscle contraction capacity occurs immediately, without requiring adaptation over time. In contrast, the findings of the present study showed that the masseter muscle thickness increased in stages from baseline to six months. The increase from initial to 6 months rates can be attributed to enhanced chewing function following rehabilitation. Based on the literature, this follow-up period was chosen because a neuromuscular adaptation was observed six to twelve months later.

It is expected that both sides of the muscle work similarly when evaluating masticatory function. Bilateral symmetric chewing is important for the proper stimulation of the supporting structures [39]. Wilding et al. reported that bilateral chewing increased chewing efficiency [40]. Zhu et al. compared three groups of patients who used removable prostheses, implant-supported removable prostheses, and implant-supported fixed prostheses to examine the effects of dental implant rehabilitation on masticatory function [41]. The study showed that implant therapy increased masticatory efficiency. In addition, the masticatory muscle asymmetry index was found to be significantly lower in the implant-supported fixed dental prosthesis group) than in the traditional removable dental prosthesis group and the implant-supported removable dental prosthesis group at 17 months of follow-up. In accordance with this, the current study also supports that the asymmetry differences between muscle thicknesses decrease significantly over time both inter- and intra-groups. This inter-group decrease was already significant at the 1st month for the masseter muscle while the temporal muscle differences could be detected at the 6th month. This can be explained by the same neuromuscular mechanism as muscle thickness increases. The intra-group analysis showed a significant baseline asymmetry between the muscle thicknesses at the test group which can be explained by malocclusion/malfunction due to tooth loss, as the asymmetry between the muscle thicknesses decreased after the denture was placed. Mandibular angle values and facial appearance are significantly influenced by the shape, function, and thickness of the masticatory muscles [42, 43]. Asymmetry of the masseter muscle can cause one side of the jaw to appear larger or more prominent than the other. This can negatively affect facial aesthetics and this condition can negatively impact an individual’s self-esteem and social life. The reduction of asymmetry between the masseter muscles also positively affects facial morphology. This indirectly and positively enhances the quality of life of patients who wear prostheses.

The age of the patient and the duration of edentulism exert a detrimental impact on the macro- and microscopic architecture of the masticatory muscles [44]. Considering this effect, to ensure standardization, the study included patients who had been edentulous least 3 months and maximum 6 months of total edentulism and had never used a prosthesis before within a similar age group.

Previous research has demonstrated the accuracy of ultrasonic imaging in evaluating masseter muscle thickness [2, 21, 23]. Ultrasound is better than other imaging methods for examining the musculoskeletal system because it has high resolution, can produce dynamic images, and allows for real-time comparisons [45]. Therefore, ultrasound imaging was preferred in this study.

The study is limited by its small sample size and its relatively short evaluation period. In addition, although the examiner was calibrated, the measurements may be influenced by technique-dependent factors such as the pressure exerted on the muscle by the probe, the probe’s angulation, and the correct definition of the muscle image. Furthermore, although patients with a history of bruxism and clenching were excluded, it is almost impossible to detect these disorders before the prostheses are placed in edentulous individuals. This situation may have modified the actual muscle thickness measurements. The findings of the study show the effect of implant-supported fixed prosthesis treatment on masticatory muscles in edentulous patients. However, these data should be supported by further studies with a larger sample size, longer evaluation periods, different implant types/numbers/locations, different prosthesis types and different novel imaging techniques.

## Conclusion

The implant-supported fixed prostheses significantly increase the thicknesses of the masseter and anterior temporal muscle together with a decrease in the asymmetry between the left and right muscles. At six months, implant-treated patients showed similar muscle thicknesses compared to dentate individuals.

## Data Availability

No datasets were generated or analysed during the current study.
